# Label-free fluorescence lifetime imaging for rapid discrimination of high-grade prostate cancer in fresh biopsy cores: a feasibility study

**DOI:** 10.1117/1.JBO.31.3.036001

**Published:** 2026-03-02

**Authors:** Julien Bec, Xiangnan Zhou, Yash Tipirneni, Shuai Chen, Jinyi Qi, Kenneth A. Iczkowski, Marc Dall’Era, Laura Marcu

**Affiliations:** a University of California, Davis, Department of Biomedical Engineering, Davis, California, United States; b University of California, Davis, Division of Biostatistics, Department of Public Health Science, California, United States; c University of California, Davis Medical Center, Department of Pathology, Sacramento, California, United States; d University of California, Davis Medical Center, Department of Urologic Surgery, Sacramento, California, United States; e University of California, Davis, Department of Neurological Surgery, Sacramento, California, United States

**Keywords:** fluorescence lifetime imaging, prostate cancer, optical biopsy, Gleason grading, tissue characterization, fiber-optic spectroscopy

## Abstract

**Significance:**

Prostate biopsy remains the gold standard for prostate cancer (PCa) diagnosis and treatment planning. However, current techniques suffer from low cancer detection rates, with most biopsy cores sampling benign tissue, leading to undergrading and repeat procedures. Label-free fluorescence lifetime imaging (FLIm) offers a potential solution by enabling real-time discrimination between malignant and benign tissue during biopsy collection, potentially reducing both the number of cores required and the repeat biopsy rates.

**Aim:**

This pilot study evaluates the feasibility of label-free FLIm for rapid discrimination of malignant from benign prostate tissue in freshly obtained core needle biopsies.

**Approach:**

Twenty patients undergoing prostate biopsy were enrolled. FLIm measurements were performed immediately after sample collection (∼10  s) using a custom fiber-optic probe. For each point measurement, FLIm parameters from four spectral bands associated with the emission of distinct endogenous fluorophores including structural proteins and metabolic cofactors (e.g., NADH and FAD) were entered in the analysis. Each FLIm point measurement was labeled based on histological annotation. These data were analyzed to characterize tissue-type differences and to train and evaluate support vector machine (SVM) classifiers for malignancy detection.

**Results:**

Separation between benign tissue and Gleason pattern ≥4  PCa can already be observed using just 2 out of 56 FLIm-derived parameters. The SVM classifier, using all parameters, achieved a receiver operating characteristic of 0.88 for identifying Gleason pattern 4 PCa. A shorter lifetime value observed in the NADH-associated band was observed for Gleason pattern 4 PCa relative to benign tissue, consistent with increased free NADH from upregulated glycolysis, supporting the biochemical basis for optical differentiation.

**Conclusions:**

FLIm demonstrates strong potential for identifying high-grade PCa. Because measurements were performed using a single fiber optic, this approach can be readily integrated into standard prostate biopsy devices to enable FLIm-guided and real-time tissue characterization during the biopsy procedure and to inform targeted tissue collection.

## 1 Introduction

### 1.1 Prostate Cancer (PCa) Is One of the Most Common Cancers Affecting Men

PCa is the second leading cause of cancer death in men worldwide.^1^ In 2018 alone, over 1.2 million new cases of PCa were diagnosed globally, with ∼359,000 deaths.^2^ PCa screening typically involves a prostate-specific antigen blood test and a digital rectal exam. If either of these is abnormal, additional testing such as another biomarker (e.g., Prostate Health Index and 4Kscore) or multiparametric MRI (mpMRI) for further risk stratification may be used to decide if a prostate biopsy is recommended.

### 1.2 Role and Limitations of Prostate Biopsy (Bx)

Accurate determination of PCa stage and Gleason pattern (GP) is essential for treatment planning. Despite performing ∼1 million prostate biopsies annually in the United States,^3^ diagnostic efficacy remains limited, with standard 12-core biopsies having up to 20% false-negative rates,^4^ and 40% risk of Gleason undergrading^5^ and 90% of biopsy cores are benign. Although pre-biopsy MRI improves yield, targeting of PCa tissue remains challenging.^6^^,^^7^ MRI–ultrasound fusion technologies require additional equipment^8^^,^^9^ without demonstrating significant benefits over cognitive fusion methods.^10^ These limitations persist even in MRI-guided biopsies,^11^ suggesting intratumor heterogeneity^12^ significantly impacts accuracy.^7^ The main factors limiting sensitivity include small sample size relative to total prostate volume, the multifocal nature of PCa,^13^ and sparse sampling, which can lead to underestimating tumor grade.

### 1.3 Label-Free Optical Imaging Technology for Prostate Biopsy Guidance

Label-free optical technologies show promise for prostate biopsy guidance by providing real-time tissue assessment without contrast agents. They can detect biochemical (e.g., fluorescence and reflectance spectroscopy^14^^,^^15^), structural (optical coherence tomography^16^^,^^17^), or cellular architecture (confocal endomicroscopy^18^) differences between benign and malignant tissues. These techniques also have the potential to be implemented via fiber optics for tissue identification directly at the biopsy needle tip. A major challenge remains: achieving reliable tissue discrimination while maintaining the size and sampling efficiency of standard biopsy devices.

### 1.4 Fluorescence Lifetime Imaging (FLIm) for PCa Detection and Characterization

Label-free FLIm offers a promising approach for cancer detection by capturing changes in extracellular matrix composition^19^^,^^20^ and cellular metabolism,^21^^–^^23^ which occur during malignant transformation. These changes lead to variations in optical signature across multiple wavelengths, enabling a comprehensive characterization of neoplastic tissue, potentially enhancing diagnostic accuracy and biological understanding of PCa progression.

In this pilot study involving freshly biopsied prostate specimens from 20 patients, we investigated whether biochemical and metabolic changes among tissue types (i.e., cancer, dysplasia, and healthy) produce distinct autofluorescence signatures detectable by FLIm. We developed both binary and multi-class classifiers to assess the preliminary efficacy of FLIm in identifying PCa tissue. This work serves as an initial step toward demonstrating the feasibility of real-time FLIm-based prostate cancer detection and guiding the future development and clinical validation of an integrated FLIm-enabled biopsy device.

## 2 Materials and Methods

### 2.1 Study Population and Design

This study, approved by the UC Davis Institutional Review Board (IRB# 222924), was performed between May and August 2024 on a cohort of 20 patients undergoing transperineal prostate Bx. Eligible patients were offered to enroll in this study. No additional biopsies were collected specifically for this study.

### 2.2 Pulse-Sampling FLIm System

**Fig. 1:**
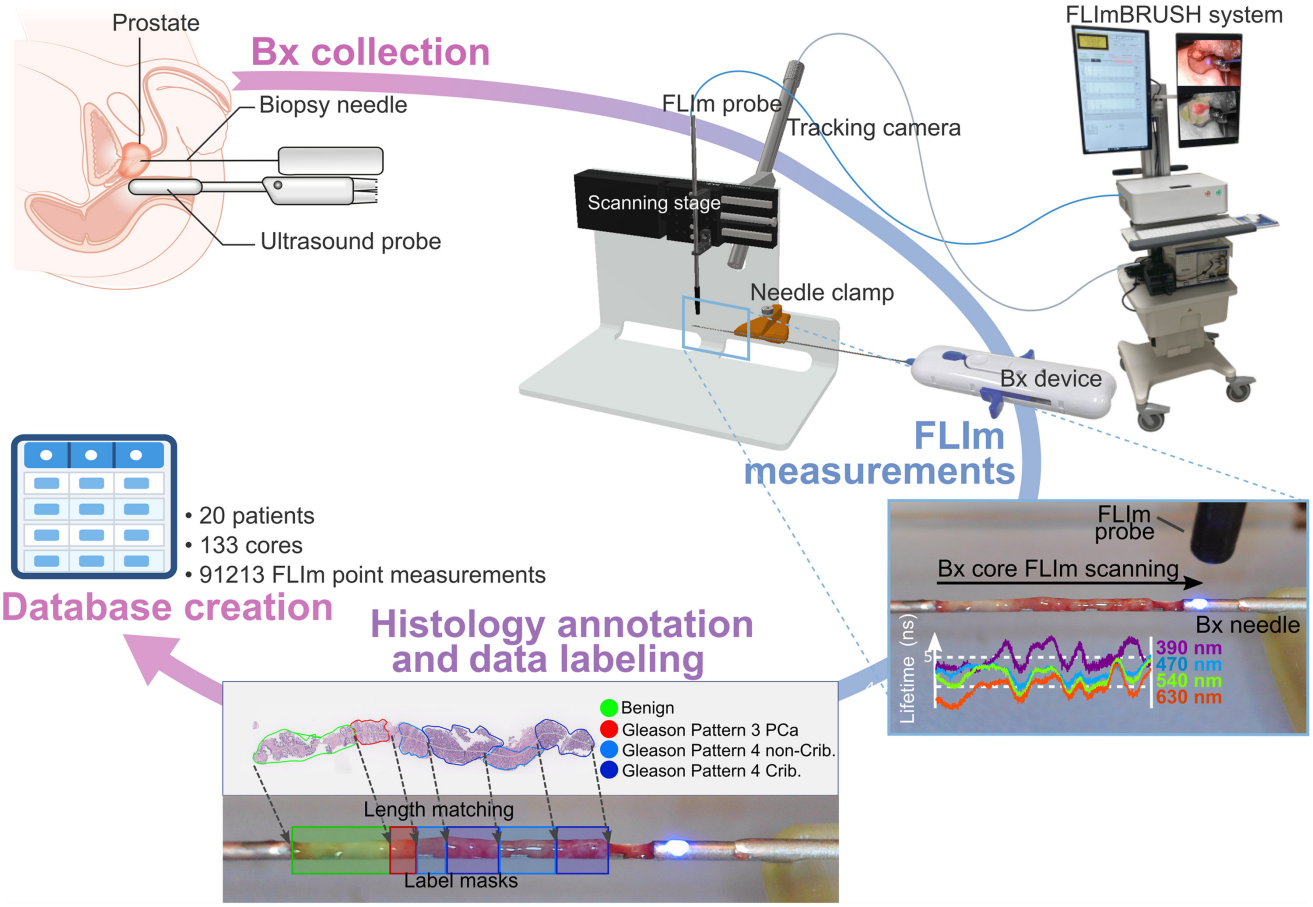
Study synopsis: transperineal prostate biopsy collection. FLIm measurements were performed along the length of the core (2.5  mm/s) using a custom automated sample scanner to ensure consistent, high-SNR measurements over the entire length of the biopsy core. Histology slide annotation and data labeling. Creation of a clinical database matching FLIm signature and tissue type.

The pulse-sampling FLIm technique employed for this work is a single-shot technique where full fluorescence decays are acquired simultaneously in multiple spectral bands in response to one excitation pulse. Figure 1 illustrates the custom-built, fiber-based, point-scanning FLIm system that consists of a console and reusable fiber optic probe. In-depth characterizations of this instrument and specifications are detailed extensively elsewhere.^24^^–^^28^ Briefly, the fiber optic probe consists of a 365-μm core multimode optical fiber terminated with a 2.5-mm ball lens, leading to an expected lateral resolution of 400  μm. It is used to deliver 355-nm pulsed UV laser excitation light to biological tissue (460-Hz repetition rate). The same optical fiber is used to relay autofluorescence point measurements from the tissue regions evaluated to the FLIm system. The fiber’s proximal collection end is coupled to a wavelength selection module which features a set of four dichroic mirrors and bandpass filters selected to capture emissions of key biological fluorophores (i.e., 390±20  nm: collagen, proteoglycans; 470±14  nm: NAD(P)H; 542±25  nm: FAD; 629±26.5  nm: porphyrins).^21^ The optical signal from each spectral band is detected by an avalanche photodiode detector and digitized (2.5  GS/s). This pulse sampling FLIm approach is suitable for use in the presence of external illumination such as operating room lights.^27^^,^^28^

### 2.3 Prostate Biopsy Sample Collection and Fluorescence Lifetime Data Acquisition

Specimens were collected during targeted biopsies under trans-rectal ultrasound guidance using an 18-G biopsy device able to collect samples of up to 19 mm length (SUREcore^®^ Plus, Uro-1, Greensboro, North Carolina, United States). Once tissue was collected, the external cannula was retracted to expose the specimen, the needle of the biopsy device was positioned and secured on the scanning fixture (Fig. 1), and FLIm measurements were performed, all within seconds (5 to 15 s) of specimen collection. Samples were not processed in any way before measurements (e.g., rinsing of blood) to account for realistic measurement conditions. Dehydration was controlled by measuring samples seconds after collection. A bidirectional scan provided repeat measurements of the core (total scan duration: 20 s) to assess FLIm measurement variability and evaluate potential changes of FLIm signature within seconds of specimen collection.

### 2.4 FLIm Data Processing

**Fig. 2:**
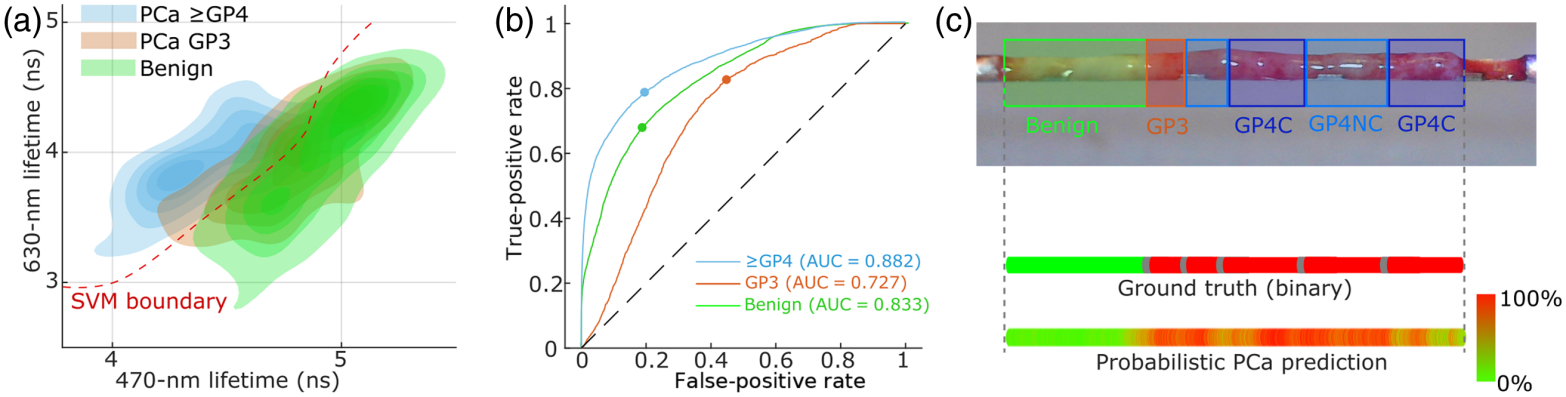
Multivariate and classification results. Contour line plot of the average lifetime signature for 470- and 630-nm bands, for benign tissue, PCa Gleason pattern 3, and PCa Gleason pattern ≥4. Overlap between benign and PCa Gleason pattern 3 observed on two-parameter discrimination is resolved using additional parameters. The SVM boundary corresponds to the PCa Gleason pattern ≥4 and benign class separability. ROC curves for three-class SVM predictor results using four average lifetimes. Illustration of a two-class (PCa, benign) probabilistic classifier output demonstrating good agreement with the original labels determined from histological evaluation.

The FLIm waveforms which originate from each spectral band were averaged four times before further processing, resulting in point measurements every 22  μm. The fluorescence decay parameters from each FLIm point measurement were retrieved using a constrained least-squares deconvolution with the Laguerre expansion method.^26^ This approach provides average lifetime values for each spectral band and captures the complex decay dynamics. For each of the four spectral bands of the instrument, the average lifetime value, spectral intensity, and 12 fitting coefficients of the Laguerre expansion were computed [Fig. 2(c)]. This leads to a total of 52 parameters (4 average lifetimes and 48 Laguerre expansion metrics). For this study, FLIm data were processed offline, but real-time data analysis can be readily implemented using our previously reported method.^27^

### 2.5 Histopathologic Processing and Annotation

Following FLIm interrogation, Bx specimens were placed on coreCARE (Uro-1, Greensboro, North Carolina, United States) specimen retrieval substrates, which are designed to preserve tissue integrity and orientation during the biopsy process. The samples were formalin-fixed, paraffin-embedded, sectioned, and stained (hematoxylin and eosin). High-resolution digital images of the stained cores were captured using the Aperio platform. Areas corresponding to different Gleason patterns were annotated by a subspecialty-trained urologic pathologist, blinded to the FLIm data (Table 1 and Fig. S1 in the Supplementary Material).

**Table 1:** Participants’ clinicopathologic characteristics.

Variable	Result
Number of patients	20
Total cores	133
Age	68.5 ± 7.9
ISUP Grade Group	
Benign	N=5
GG1	N=4
GG2	N=5
GG3	N=4
GG4	N=1
GG5	N=1
Tumor GP distribution (number of FLIm point measurements/number of patients in which it is present)	
Benign (prostatic tissue, fibromuscular stroma, seminal vesicle, atrophic benign gland, and high-grade prostatic intraepithelial neoplasia)	N=80,614/N=20
GP3	n=3936/N=12
GP4, not cribriform	n=4562/N=5
GP4 with cribriform	n=2012/N=4
GP5	n=89/N=1

### 2.6 Database Creation

FLIm data were labeled by co-registering the FLIm measurement position along the fresh core with the annotated histologic sections. Differences in length between fresh cores and slides of the core, attributed mainly to stretching and shrinking during fixation and processing, were addressed by assuming a uniform deformation of the specimen. In addition, 25-point measurements at each label transition, corresponding to 0.54 mm, were excluded from the dataset to reduce the inclusion of mislabeled data. The complete study database consisted of 91,213 FLIm points obtained from 133 cores from 20 patients and included labels as well as patient-level demographic and diagnostic information.

### 2.7 Data Analysis and Machine Learning

Measurement repeatability was evaluated across the entire dataset by systematically evaluating differences in average lifetime for each spectral band among repeat measurements. Differences among consecutive measurements of the same specimen may originate from variability in FLIm measurements as well as potential changes in the fluorescence signature at the sample level. Variation in signal with frequency higher than the spatial resolution of the system can be attributed to measurement noise; thus, an 18-point (equivalent to 0.4 mm) moving average was applied to lifetime traces of each repeat scan (Fig. S2 in the Supplementary Material). The measurement noise intensity was computed as the standard deviation of the difference between original and smoothed traces, and the measurement noise in the smoothed traces was estimated by dividing the original noise by the square root of the number of points used for averaging. The mean absolute error (MAE) among smoothed repeat scans was calculated and compared with three times the estimated noise standard deviation in the smoothed traces. When the MAE exceeded this threshold, the difference among repeat measurements was considered statistically significant with a 99% confidence interval, indicating a genuine change in FLIm signature rather than measurement variability. To assess a potential effect on tissue classification performance, we compared the MAE among repeat scans to the standard deviation of the original measurement noise.

Multivariate analysis was initially applied to qualitatively evaluate whether FLIm-derived parameters could distinguish among tissue types. This preliminary examination focused on identifying whether one or more parameters showed potential differentiation capabilities, serving as a first-pass screening before more rigorous statistical validation. Visual inspection of parameter distributions suggested that even a minimal subset of FLIm features might effectively separate tissue categories, guiding subsequent quantitative analyses (Fig. 2). A support vector machine (SVM) boundary computed over the entire dataset was used to evaluate the separability of G≥4 tissue signature and healthy tissue [Fig. 2(a) and Table 2, basic separability].

**Table 2:** Classification performance for different tissue discrimination approaches.

Discrimination	Basic separability	Two-class prediction		Three-class prediction	
Classification model	Support vector machine				
Tissue class	• PCa GP4+ • Benign	• PCa (GP3 + GP4 + GP5) • Benign	• PCa (GP4 + GP5) • PCa GP3 + benign	• PCa (GP4 + GP5) • PCa GP3 • Benign	• PCa (GP4 + GP5) • PCa GP3 • Benign
Validation approach	No separate training/validation sets	Leave-one-patient-out cross-validation			
Parameters	• 470-nm LT • 630-nm LT	• 390-nm LT • 470-nm LT • 540-nm LT • 630-nm LT	• 390-nm LT • 470-nm LT • 540-nm LT • 630-nm LT	• 390-nm LT • 470-nm LT • 540-nm LT • 630-nm LT	LT and Laguerre coefficients for all spectral bands (n=52 parameters)
ROC-AUC	0.950	0.835	0.887	Benign: 0.833 95% CI [0.788, 0.867]. GP3: 0.727 95% CI [0.596, 0.767]. GP4+: 0.882 95% CI [0.847, 0.941].	Benign: 0.772 GP3: 0.711 GP4+: 0.789
Sensitivity/specificity	86.4%/90.1%	84.4%/62.9%	79.3%/85.0%	Benign: 68.0%/81.2% GP3: 82.7%/55.2% GP4+: 78.8%/80.5%	Benign: 67.2%/73.5% GP3: 82.3%/50.9% GP4+: 71.2%/71.2%

To assess whether FLIm data could effectively discriminate between healthy and PCa tissue, we evaluated SVM using average lifetimes from individual bands as well as the entire set of parameters obtained from the Laguerre deconvolution approach. Binary SVM classifiers were constructed using a Gaussian (radial basis function) kernel. A fixed box constraint (regularization parameter) of 0.1 was used to control the trade-off between margin maximization and classification error. A leave-one-patient-out (LOPO) approach was implemented to prevent data leakage. Receiver operating characteristic area under the curves (ROC-AUCs) were computed by aggregating class probability predictions across all patients and generating a single ROC curve from the pooled data.

The number of point measurements varies across patients, due to the number and length of biopsy cores; thus, a patient-level weighting based on the number of points was applied to the overall ROC-AUC computation to ensure that each patient is represented equally. Patient weighting was not applied when determining SVM hyperplanes, but class imbalance was addressed by assigning different misclassification costs inversely proportional to the frequency of each class in the training.

Three-class classification was performed using an SVM framework with error-correcting output codes (ECOCs), as implemented in MATLAB. Binary SVM learners were constructed for each ECOC partition. The ECOC strategy decomposed the multiclass problem into multiple binary classification tasks, improving robustness and scalability when handling more than two classes. Model training was conducted on the feature vectors extracted from the imaging data, and classification performance was evaluated using leave-one-patient-out cross-validation. This multiclass SVM-ECOC approach enabled effective discrimination among multiple tissue classes while mitigating overfitting in high-dimensional feature spaces.

To estimate the confidence interval of the three-class SVM ROC-AUC, a patient-level bootstrap procedure with 100 replicates was applied by random resampling patients with replacement at the fold level, preserving within-patient correlations. For each bootstrap replicate, LOPO-CV was repeated, and an AUC value was obtained. The empirical distribution of bootstrap AUCs was used to derive the 95% confidence interval, defined by the 2.5th and 97.5th percentiles.

The consistency of the prediction was evaluated across different classifier types. Three-class prediction using four average lifetime values was also assessed using linear discriminant analysis (LDA), quadratic discriminant analysis (QDA), logistics regression (LR), and decision trees (DTs).

Finally, to evaluate the effect of data leakage on classification performance, as observed in earlier publications,^14^ we also evaluated classification performance using a 10-fold cross-validation where folds were randomly determined irrespective of patients.

## 3 Results

### 3.1 Measurement Repeatability

The repeatability analysis demonstrated excellent consistency across sequential FLIm measurements. Quantitatively, only 3.3% of all scan points showed statistically significant differences among repeat measurements when evaluated at a 99% confidence threshold [Fig. S2(b) in the Supplementary Material]. This degree of consistency was maintained across all four spectral bands, underscoring the robustness of measurement stability. Furthermore, for all but a single isolated case (involving a single spectral band in one scan), the magnitude of variation remained within the inherent noise level of the system [see Fig. S2(c) in the Supplementary Material]. This high level of repeatability confirms that variations in FLIm signature during the brief measurement period are minimal and therefore unlikely to introduce confounding factors that would adversely affect tissue classification performance.

### 3.2 Multivariate Analysis

Examination of the FLIm parameter space revealed distinct clustering patterns among tissue types. Most notably, benign prostatic tissue and Gleason pattern 4 PCa demonstrated clear separation using only two parameters, for example, the 470- and 630-nm lifetime values [Fig. 2(a)]. This separation is particularly significant as it indicates that high-grade cancer can be distinguished using a minimal set of FLIm parameters. In contrast, Gleason pattern 3 cancer tissue demonstrated considerable overlap with both benign and Gleason pattern 4 signatures when using two parameters, suggesting intermediate biologic characteristics that are reflected by the FLIm measurements. When focusing specifically on Gleason pattern 4 patterns, no distinct differences were observed between cribriform and non-cribriform subtypes using our current parameter set. Quantitatively, applying an SVM classifier to the entire dataset based solely on these two parameters yielded an ROC-AUC of 0.95 for distinguishing Gleason pattern 4 PCa from benign tissue (Table 2), demonstrating the strong diagnostic potential of this approach even with a simplified parameter set.

### 3.3 Tissue Predictor Performance

Table 2 provides a comprehensive comparison of SVM classification performance across multiple tissue discrimination scenarios and parameter combinations. Although the dataset contains ∼90,000 FLIm point measurements, these observations are tightly clustered along biopsy tracks and therefore are not statistically independent. Our leave-one-patient-out cross-validation ensures that measurements from a given patient do not contribute to both training and test folds, which mitigates the data leakage issue. However, after taking into account the spatial sampling (22  μm) and lateral resolution of the fiber optic probe (0.4 mm), the effective sample size is ∼5% of the size of the original dataset. More comprehensive modeling of the hierarchical data structure (e.g., mixed-effects or core-level analyses) will be important in future studies with larger cohorts. Consistent with the multivariate analysis findings, the highest classification accuracy was achieved when discriminating Gleason pattern ≥4  PCa from benign and Gleason pattern 3 tissue (ROC-AUC: 0.887), whereas Gleason pattern 3 cancer showed more limited separability due to its intermediate FLIm signature characteristics. This performance hierarchy aligns with the biologic continuum from benign tissue to increasingly aggressive cancer phenotypes. Notably, we found that using just four average lifetime parameters (one from each spectral band) outperformed a more complex SVM model that utilizes the full set of 52 lifetimes and Laguerre expansion parameters. This finding suggests that the larger model may be prone to overfitting given the limited dataset, whereas the reduced parameter set offers more robust and generalizable classification performance. On the other hand, classifiers with additional parameters, combined with a more expansive dataset, may help with the discrimination of Gleason pattern 3 or 4 cribriform versus Gleason pattern 4 non-cribriform. Classification performance for three-class prediction using four average lifetimes, performed using LDA, QDA, LR, and DT classifiers, is reported in Fig. S3 in the Supplementary Material. When comparing validation methodologies, we observed that the conventional 10-fold cross-validation approach (which does not respect patient boundaries) produced an artificially inflated discrimination performance (ROC-AUC: 0.97), further validating our decision to implement the more rigorous leave-one-patient-out validation strategy to obtain realistic performance estimates.

## 4 Discussion

This work establishes FLIm as a promising approach for real-time label-free discrimination of PCa tissue, with the strongest performance associated with the identification of Gleason pattern ≥4  PCa, which represents a major threshold between less aggressive and more aggressive disease.

### 4.1 Diagnostic Performance Implications

Multivariate analysis revealed consistent, diagnostically relevant contrast between benign and cancerous prostate tissue, with the highest discrimination observed between Gleason pattern ≥4  PCa and benign tissue. The observed overlap among tissue type distributions likely stems partially from registration challenges between fresh specimens and histological sections, establishing a practical upper limit to achievable classification accuracy. Nevertheless, our classifier demonstrated robust performance in distinguishing PCa from benign tissue (ROC-AUC: 0.833) and particularly in identifying high-grade PCa (ROC-AUC: 0.88). Evaluation of different classifiers to the three-class classification showed that SVM and QDA achieved the highest diagnostic performance, whereas LDA, logistic regression, and decision trees yielded somewhat lower but still reasonable classification accuracy (AUC range for GP4: 0.865 to 0.814), indicating that the fluorescence lifetime features enable effective tissue discrimination across a range of algorithmic approaches, from simple linear classifiers to more complex non-linear methods. The ability to specifically detect Gleason pattern ≥4  PCa has substantial clinical relevance because more than minimal percentages of high-grade tumor usually render a patient ineligible for active surveillance. By providing real-time identification of high-grade cancer during the biopsy procedure, FLIm technology may improve targeting of clinically significant cancer, enhancing both diagnostic accuracy and treatment planning.

### 4.2 Biological Basis of FLIm Contrast

The diagnostic potential of FLIm stems from its ability to capture simultaneously two fundamental biological changes in cancerous tissue. First, structural remodeling of the extracellular matrix occurs both within tumors and their surrounding microenvironment,^19^^,^^20^ producing detectable changes in the 390-nm emission band through altered collagen and proteoglycan fluorescence signatures. Second, cancer cells undergo characteristic metabolic reprogramming through the Warburg effect—prioritizing glycolysis and lactate production even in oxygen-rich environments while reducing dependence on oxidative phosphorylation. This metabolic shift disrupts the equilibrium between free and protein-bound forms of NADH and FAD,^21^^–^^23^ creating distinctive fluorescence lifetime patterns. Our data demonstrate this metabolic shift through the decreased fluorescence lifetime observed in the 470-nm band of tumor tissue, consistent with elevated levels of free NADH typical of glycolytic metabolism. Complementary metabolic information is captured in the 540-nm band through FAD fluorescence variations, whereas the 630-nm band detects changes in lipofuscin, an age-related pigment previously linked to PCa’s optical signature.^14^ This multi-spectral approach enables comprehensive characterization of prostate tissue through simultaneous assessment of both structural and metabolic alterations associated with malignancy.

### 4.3 Translational Relevance of *Ex Vivo* Findings

A major strength of our approach was acquiring measurements within seconds of biopsy collection. This was made possible by placing the instrumentation directly in the procedure room and implementing a rapid measurement protocol that preserved tissue integrity. This represents a methodological improvement over prior studies^14^^,^^29^ on prostatectomy specimens, where tissue was imaged an hour or more after blood supply termination—conditions likely to alter metabolic fluorescence signatures. The consistency of FLIm parameters observed in our repeat measurements (separated by up to 25 s) provides strong evidence that the optical signatures remain stable during the brief interval between specimen collection and measurement. This temporal stability suggests that the FLIm signatures we observed are likely representative of *in vivo* tissue characteristics.

### 4.4 Methodological Improvements over Prior Work

Several aspects of our study design address key limitations identified in prior reports. Unlike earlier studies^14^ that focused on predefined regions of interest, our analysis incorporated point-by-point measurements along the entire length of biopsy cores, enabling a more comprehensive characterization of tissue heterogeneity.^14^ In addition, we implemented patient-level separation in our validation strategy to overcome a common methodological limitation of traditional K-fold cross-validation, which, when applied at the point level, can result in training and testing on highly correlated measurements from the same patient. Such correlation among spatially adjacent measurements artificially inflates reported performance metrics and compromises model generalization. By employing a “leave-one-patient-out” cross-validation approach, our analysis provides a more realistic estimate of clinical performance, further supported by the consistency of results across multiple classification algorithms and parameter combinations.

### 4.5 Study Limitations and Future Work

Although our cohort of 20 patients was sufficient to demonstrate preliminary efficacy, validation in a larger and more diverse patient population will be necessary to establish definitive classification performance. Moreover, although our *ex vivo* measurements showed remarkable consistency with minimal temporal variation, direct confirmation through comparative *in vivo* studies will be essential to fully validate the translational potential of this approach. Future efforts focus on integrating FLIm technology directly into biopsy instrumentation which will enable real-time tissue characterization during clinical procedures.

## 5 Conclusion

Although future work will be required to integrate FLIm technology directly into biopsy instrumentation for real-time guidance of biopsy procedures, this work provides evidence of preliminary efficacy for real-time PCa detection in *ex vivo* specimens to justify further technology development. Our findings demonstrate that FLIm can reliably differentiate clinically significant (Gleason pattern ≥4) PCa from benign tissue (ROC-AUC: 0.883) by detecting intrinsic metabolic and extracellular matrix composition. The single-fiber design is inherently compatible with existing biopsy instruments, offering a clear pathway toward guided procedures that improve diagnostic yield. Although further validation in larger cohorts is warranted, these results suggest that FLIm technology could meaningfully advance PCa diagnosis, inform treatment planning, and ultimately improve patient outcomes.

## Supplementary Material

10.1117/1.JBO.31.3.036001.s01

## Data Availability

The data and associated data analysis code that support the findings of this study will be made available through the National Center for Interventional Biophotonic Technologies upon reasonable request. Due to the nature of this research involving human participants, data sharing is subject to appropriate data use agreements and compliance with institutional review board requirements to protect patient privacy.
