# Supplementary material for: Label-free fluorescence lifetime imaging for rapid discrimination of high-grade prostate cancer in fresh biopsy cores: a feasibility study
Source: J Biomed Opt. 2026 Mar 2;31(3):036001. doi: 10.1117/1.JBO.31.3.036001 (PMC12950611; doi:10.1117/1.JBO.31.3.036001)
Supplement: Supplementary file 1 [file JBO_031_036001_SD001.pdf]

Supplementary material

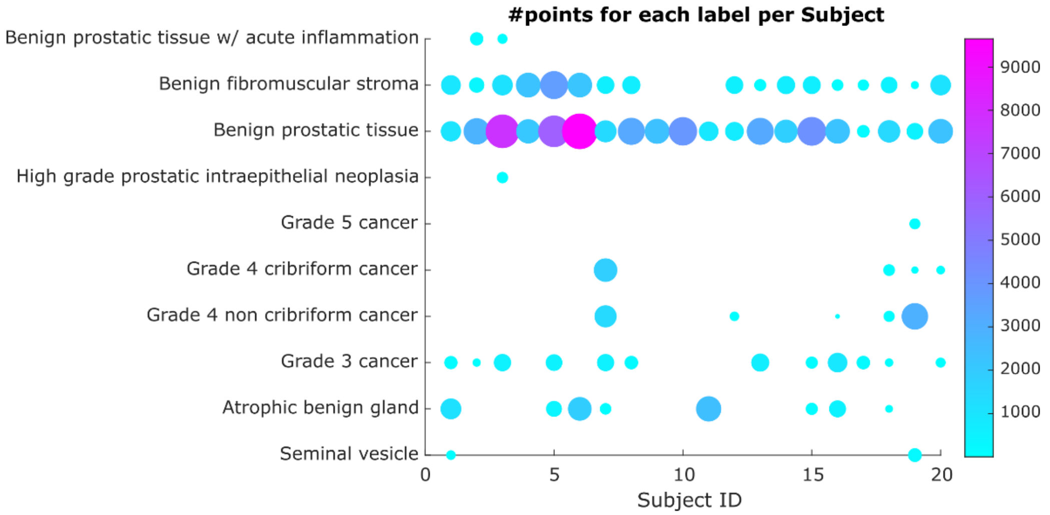

Figure S1: Representation of study database. Each dot represents the number of points for each label and each patient enrolled in the study.

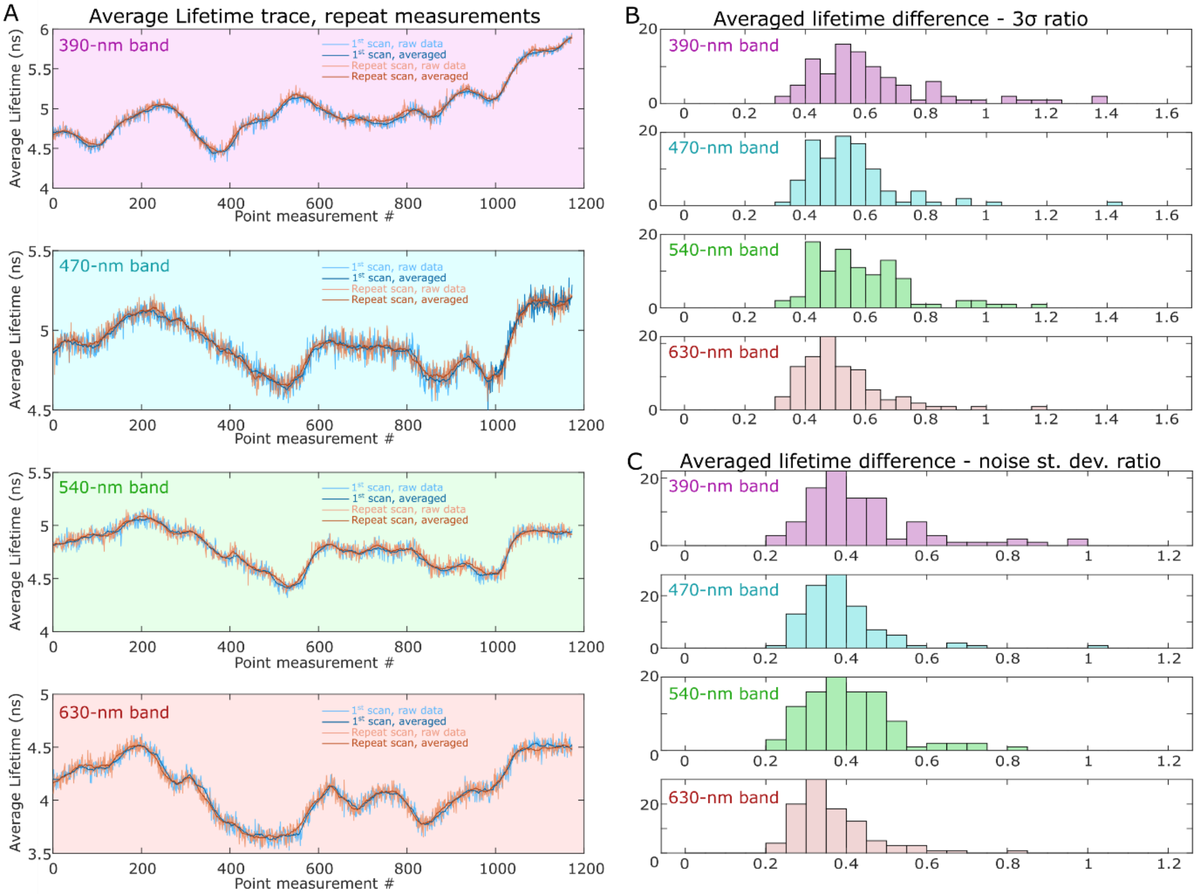

Figure S2. Measurement repeatability evaluation. Raw/smooth traces of average lifetime for the four spectral bands of the instrument (A). Ratio of mean absolute error and 3 standard deviations of the estimated noise in the smooth traces. Values above 1 show that the difference is unlikely to be due to measurement noise alone with a confidence interval of .99 (B). Ratio of mean absolute error and standard deviations of the estimated noise in the original traces demonstrating that differences in repeat scans are well below the measurement noise (C)

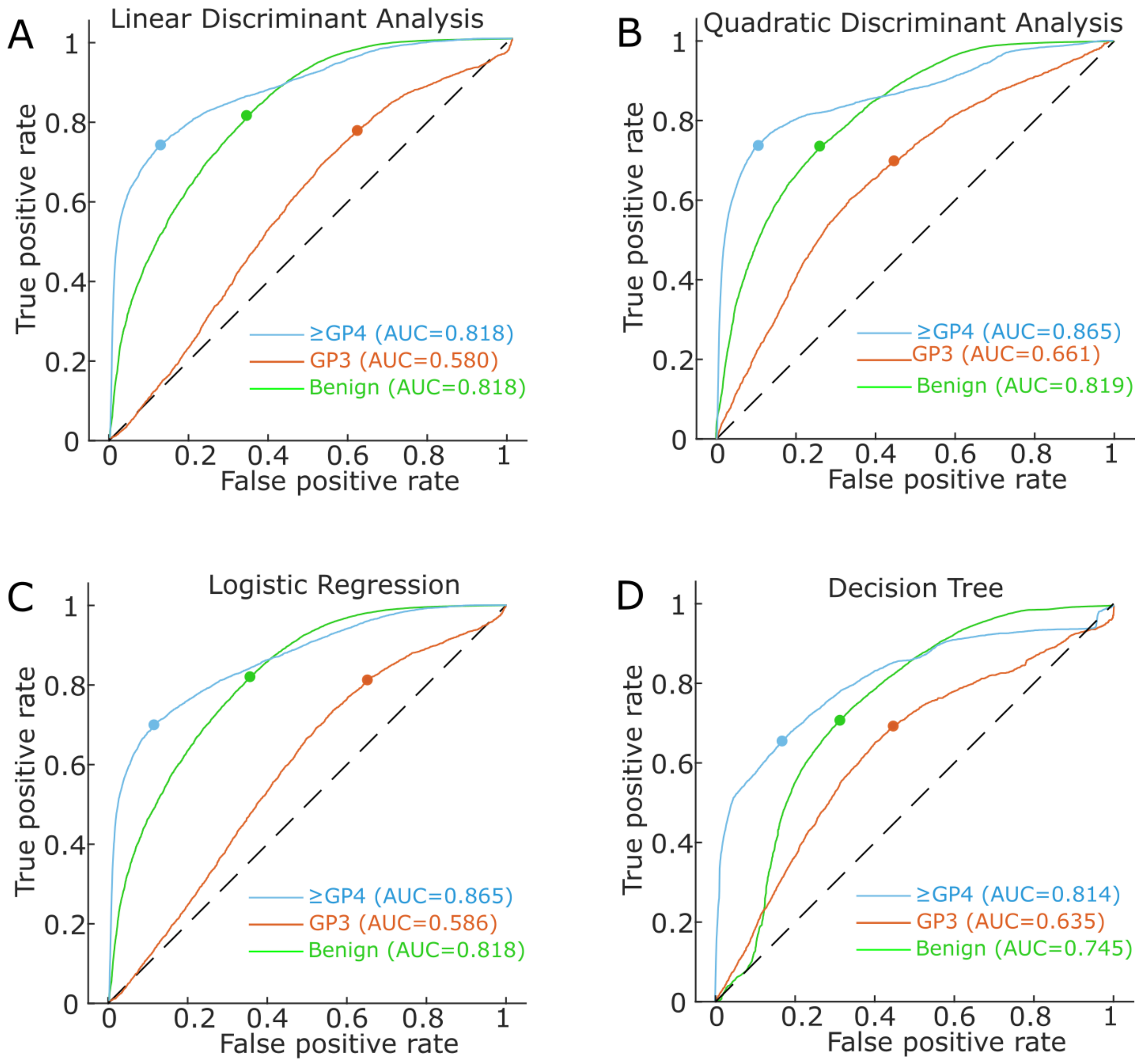

Figure S3. ROC curves for 3-class prediction using 4 average lifetimes. Linear Discriminant Analysis (A), Quadratic Discriminant Analysis (B), Logistic Regression (C), and Decision Tree (D).
